# Obituary for Professor Ian Reay Mackay (1922–2020): A pioneer Autoimmunologist

**DOI:** 10.31138/mjr.31.1.98

**Published:** 2020-03-31

**Authors:** Dimitrios P. Bogdanos

**Affiliations:** Department of Rheumatology and Clinical Immunology, Faculty of Medicine, School of Health Sciences, University of Thessaly, Larissa, Greece

It is with great sadness that I learnt of the death of Professor Ian Reay Mackay, AM, FAA, FRACP, FRCP, FRCPA on 24^th^ March 2020 at the age of 98. Professor Mackay was an Australian clinician and researcher and a true pioneer in the field of autoimmunity. His research led to a new era of autoimmune diseases, regarding their diagnosis and pathogenesis, as well as treatment with immunosuppressive drugs.

Professor Mackay was educated at the University of Melbourne, later training at Hammersmith Hospital, in London, with Dame Sheila Sherlock, a pioneer in the then-emerging field of hepatology. He was the one to introduce the term of “lupoid hepatitis”, describing what was next appreciated as chronic active hepatitis, and more recently as autoimmune hepatitis. At the laboratory of Deborah Doniach, he had the privilege to familiarize assays focused on autoimmune diagnostics. He was the first to describe the presence of an autoantibody in a 37-year-old woman with primary biliary cirrhosis, what nowadays stands as anti-mitochondrial autoantibody. He was one of the first to describe and assess the presence of autoantibodies, hypergammaglobulinaemia, tissue deposition of immune complexes, and lymphocytic and plasma cell infiltration in several autoimmune rheumatic and liver diseases.

His background knowledge on the effect of corticosteroids on the absorption of proteins and fat and the homeostasis of electrolytes in early 1950s has placed him in the unique position to understand the effect that this treatment may have on the management of patients, and comprehensively implemented immunosuppressive treatments in patients’ autoimmune hepatitis, systemic lupus erythematosus, rheumatoid arthritis, and other autoimmune diseases. The protocol he devised for treating autoimmune hepatitis with steroids is still the cornerstone for treating the disease. Back then, he extensively published several monographs in influential journals such as New England Journal of Medicine, Lancet, and British Medical Journal.

After working in the United States, in 1956 Professor Mackay has returned to Melbourne for good, collaborating with Sir Frank Macfarlane Burnet. Professor Burnet was the recipient of 1960’s Nobel Prize for Physiology or Medicine, and is best known for developing the theory of clonal selection. Burnet’s theory influenced the way research on immunology was performed, and was substantiated when Peter Medawar succeeded in performing transplants of tissue between different mouse foetuses. The results of their work influences organ transplantation management even until now. At that time, several chronic diseases were of unknown aetiology. Burnet and Mackay went a step further to propose several of those to be of autoimmune nature. They immensely influenced the scientific community when they published their seminal monograph “*Autoimmune diseases: pathogenesis, chemistry and therapy”*, written in 1961 and published one year later. It was the first publication solely on the topic of autoimmunity. This publication was considered as “a founding text” that marked the beginning of auto-immunity as a clinical science.

I considered Ian a good friend, a valued colleague and a great collaborator. I had the privilege to collaborate with him when I was in London on a project assessing the role of liver-related autoantibodies in patients with autoimmune liver diseases and autoimmune rheumatic diseases. We published that paper in 2008, and it is still one of my most highly cited papers. During the preparation of the manuscript, he drove me crazy. Ian revised my original draft so many times, it made me feel like an awful writer. I was totally frustrated. When I discussed that with my, at that time, supervisor and mentor, Diego Vergani, co-author of the manuscript and an expert on autoimmune diagnostics, I was told that this is the minimum that I can expect from Ian Mackay. He was a perfectionist. Years later I dared to ask him about my writing performances. I still remember the time Ian told me, “for a Greek, you are writing well. Greeks tend to write so much and mean so little or write so little and mean everything. Keep your writing simple and clear, like Ancient Greeks did”. I am really grateful to him.

After his retirement, Professor Mackay remained extremely active, holding a research position at Monash University, and contributed to many research collaborations. He also continued to write research publications and academic textbooks until recently.

All of us that had met and collaborated with him are extremely proud for his life, his accomplishments and his major contributions to medical science, autoimmunity in particular. We are grateful for his input to our scientific work and clinical practise. Professor Ian Reay Mackay has left a lasting legacy and will be greatly missed.

